# Women Academics’ Intersectional Experiences of Policy Ineffectiveness in the European Context

**DOI:** 10.3389/fpsyg.2022.810569

**Published:** 2022-05-06

**Authors:** Susanne Täuber

**Affiliations:** Faculty of Economics and Business, University of Groningen, Groningen, Netherlands

**Keywords:** academia, policy ineffectiveness, psychological safety, voice, intersectional inequality, intersectional privilege

## Abstract

Despite policy efforts targeted at making universities more inclusive and equitable, academia is still rife with harassment and bullying, and opportunities are far from equal for everyone. The present preregistered survey research (*N* = 91) aimed to explore whether an intersectional approach can be useful to examine the tangible effects of policy ineffectiveness, even when legislative and ideologic constraints limit the possibility to conduct a full-fledged intersectional analysis. Policy ineffectiveness was operationalized as experiences of harassment, discrimination, institutional resistance to gender equality, and retaliation against reporters of misconduct in universities. Policy ineffectiveness was negatively related to women academics’ inclination to pursue an academic career. This relationship was mediated by lower levels of psychological safety associated with policy ineffectiveness. Importantly, women academics who differ from the majority on multiple dimensions show a stronger and more negative relationship between policy ineffectiveness and psychological safety. The study further shows that self-report measures are useful to uncover intersectional privilege afforded to overrepresented groups in academia. The study discusses the benefits of intersectional approaches for designing and implementing effective policies to tackle harassment and inequality in academia, even when the available methodologies are constrained by legislation and ideology. Overall, self-report measurement can have an important function for signalling areas that warrant further intersectional inquiry to ensure that policies serve everyone.

## Introduction

*“Everybody talks about equality in science, but it does not actually happen,” … “There are so many articles, so much discussion, but over my 30 years it’s gotten worse” (quote by a researcher interviewed for a Nature survey on discrimination, cf.* Woolton, 2021).

Policies aiming to make higher education institutions more inclusive, equitable, and safe environments have been around for decades. However, scholars and practitioners are becoming more vocal about the pervasive ineffectiveness of such policies. For instance, there is growing consensus that anti-harassment and non-discrimination policies have been ineffective in making academia a more inclusive and safe working environment (Bondestam and Lundqvist, 2018, 2020; Abbott, 2020; Bull et al., 2021; Woolton, 2021; Hilton and Täuber, 2022). On the contrary, non-discrimination policies and laws have led to an epidemic of subtle and selective discrimination (Cortina et al., 2013; Jones et al., 2017). Relatedly, despite efforts to formulate concrete and implementable zero-tolerance policies (e.g., Halkitis et al., 2020), scholars criticize that such documents often end up being purely performative (Ahmed, 2007b; Kimura, 2014). For instance, in the context of fighting racism in higher education in the United Kingdom, Ahmed (2007b) observes that universities committed to the Race Relations Amendment Act “end up doing the document rather than doing the doing.”

Building on the above, in the current research, policy ineffectiveness is operationalized as women academics’ experiences of harassment and discrimination, retaliation against reporters of harassment and discrimination, as well as institutional resistance against gender equality. This operationalization covers ineffective anti-harassment and non-discrimination policies, ineffective complaints procedures, and ineffective interventions and measures to achieve gender equality. The current research explores the tangible effects of policy ineffectiveness on women in academia by examining how it appears to be related to female scholars’ psychological safety and voice. Moreover, female scholars who differ from the majority on multiple dimensions within their respective university context might be harmed by policy ineffectiveness more than their counterparts. The most influential approach for thinking about how differing from the majority on a number of dimensions affects individuals and groups is intersectional theory (Crenshaw, 1989), which I will elaborate below. Psychological safety and voice both relate to speaking up and sharing experiences, thereby enabling ineffective policies and interventions to be improved. If psychological safety and voice are undermined, a vicious circle might result where especially the most vulnerable scholars are hurt by policy ineffectiveness, diminishing their willingness to speak up for change, thereby perpetuating and reproducing working environments that undermine the effectiveness of policies.

### The European Context: Intersectionality and Data Gaps in Higher Education Policy

Before introducing the theoretical background, a reflection on the use of intersectionality in the context of European higher education policy seems useful, because it highlights a discrepancy between scholarly insight concerning intersectionality and the implementation of such insight in policy making. The concept of intersectionality was developed and introduced by legal scholar and civil rights activist Kimberlé Crenshaw (1989). She criticized the traditional accounts of feminism and antiracism, which, by focusing on White women and Black men, respectively, effectively erase the lived experiences of Black women. Crenshaw introduced intersectionality to go beyond these accounts, stating “Because the intersectional experience is greater than the sum of racism and sexism, any analysis that does not take intersectionality into account cannot sufficiently address the particular manner in which Black women are subordinated,” (1989, p. 140). Intersectionality makes visible how multiple axes of oppression interact and sheds light on unique experiences of inequality and injustice felt by people with intersecting identities (Cath et al., 2014; Atewologun, 2018). While intersectionality is important to Black American feminist intellectual heritage (Wekker, 2021), a side-effect of the great success of Crenshaw’s work is that the term intersectionality has become a “travelling concept” (Jouwe, 2015).

This results in intersectionality being used in different ways when it permeates other cultural and demographic contexts (Bowleg and Bauer, 2016; Bauer et al., 2021). Intersectionality is also used differently across disciplines, with psychological research in particular being associated with “weak” approaches to intersectionality (Grzanka, 2018). The distinction between “weak” and “strong” intersectionality is that the former focuses more on multiple identities, a core area of interest in psychological research, while the latter focuses on the co-constitution of these identities and how these are embedded in systems of power (Dill and Kohlman, 2012). Weak approaches to intersectionality often fail to investigate “the intersections of identities or the ways in which those intersections produce unique subjectivities, privilege-oppression nexuses, and lived experiences” (Grzanka, 2018, p. 20). In other words, rather than truly exploring the intersections of identities, weak approaches will yield additive insights that treat identities as if they were separate, independent and could be ranked (Bowleg, 2008). This is at odds with Crenshaws definition which highlights that intersectional experiences are greater than the sum of separate axes of oppression such as racism or sexism.

Importantly, quantitative research methods are most likely to use “weak” intersectionality (Bauer et al., 2021), but are also the most prevalent input for policy-making. This is reflected in leading publications into European higher education neglecting intersectionality, as illustrated for instance by the SHE figures (2021), which only collects data on gender. The relative neglect of intersectionality in European higher education policy appears to result from two issues in particular. First, Europe has implemented the world’s strictest data privacy law, the EU General Data Protection Regulation (GDPR). Data protection is seen as a fundamental human right (Goddard, 2017), owing to the horrors of the Second World War: the registration of names, maiden names, residence, gender, birthday, religion, mother tongue, ethnicity, occupation, and number of children in the respective household in the German census formed the bureaucratic prerequisite for the deportation and murdering of millions of Jews (Aly and Roth, 2004). Due to its specific historic context, European higher education institutions cannot register data that could reliably demonstrate structural and systemic disadvantages resulting from intersecting categories such as race, religious affiliation, and disability status. As a consequence, the option to ask scholars to self-identify their minority status in surveys is currently explored, not just in the European context (Else and Perkel, 2022).

Second, different from the US context, “Europe’s depoliticization of race and its relation to power as an analytical dimension” (Rose, 2022, p. 7) ultimately results in Black women, for instance, “being erased from projects of intersectionality despite their knowledge production and contributions” (Rose, 2022, p. 7). When more categories than gender are taken into consideration (e.g., race, ability, race, ethnicity, gender, nationality, politics, citizenship, or socioeconomic status, Perlman, 2018), the framing is often in terms of disadvantages and challenges rather than oppression and discrimination. This leads to intersectionality being used in ways that might be less threatening and more self-serving for those comparatively privileged individuals using the term—as described, for instance, by Robin Diangelo in “White fragility” (2018): where there are no oppressed, there is no oppressor, and if multiple axes of disadvantage are considered, almost everyone is a minority in some way. In Netherlands, Cath et al. (2014) refer to this approach as ‘Dutchifying intersectionality’, criticizing that the term is used as a lip-service to underrepresented groups. The authors attribute this partly to views of activism as violating academics’ objectivity, resulting in weak links between academia and activism.

The legislative and ideologic pretexts described above result in policy-making largely devoid of intersectional approaches, despite the impressive intersectional scholarship that is created and shared in Europe (e.g., Essed, 2001; Ahmed, 2007a, 2012; Özbilgin et al., 2011; Atewologun et al., 2016; Wekker, 2016; Tariq and Syed, 2017; Atewologun, 2018; Jordan-Zachery, 2019; Liu, 2019; Showunmi, 2020; Bhatti and Ali, 2021).

In addition, a systematic review of quantitative research methodologies into intersectionality from 1989 to 2020 (Bauer et al., 2021) finds that quantitative methods are often simplistic, misapplied, or misinterpreted. In light of the practical limitations with data collection outlined above, the current paper aims to explore the potential of the survey method for flagging areas that should prompt more sophisticated research to uncover intersectional inequalities. One recommendation resulting from the current paper might be to engage in a stepwise process where initially, methods are used which are suboptimal and simplistic, yet available, affordable and pragmatic. These could be instrumental for signalling areas that need to be followed up with more suitable, designed-for-purpose methodologies to uncover intersectional inequalities, such as in-depth interviews (Atewologun, 2018; Windsong, 2018). Ultimately, a stepwise approach that embraces imperfections in the initial phase might assist in designing and developing more effective policies to tackle inequalities in higher education. In sum, I investigate intersectionality here within the legislative and ideologic constraints present in the European policy context. I explore whether women scholars’ self-reported minority status on a variety of axes—reflecting an additive approach to intersectionality—might fulfil a signalling function regarding policy ineffectiveness and its relation to women scholars’ psychological safety, voice, and career choices.

## Theoretical Background

### Harassment and Policy Ineffectiveness

As disciplines, the social and organizational sciences have the means and capacities to understand, analyse, and describe phenomena that undermine gender equality in higher education, such as discrimination and harassment. However, we appear less well equipped to practice what we preach: like other organizations, universities fail to live up to their expressed egalitarian and social justice goals (e.g., Naezer et al., 2019; Abbott, 2020). Academia has the second-highest rate of reported sexual harassment (in comparison with military, which has the highest rate, the private sector, and government; Ilies et al., 2003). Accordingly, harassment and bullying are described as epidemic in academia (Mahmoudi, 2019, 2020; Gewin, 2021; Moss and Mahmoudi, 2021; Täuber and Mahmoudi, 2022) and retaliation against reporters of misconduct is a key contributing factor (Bergman et al., 2002; Cortina and Magley, 2003).

In the social sciences, sexual harassment is used as an umbrella term comprised of unwanted sexual attention, sexual coercion, and gender harassment (Cortina and Areguin, 2021). While unwanted sexual attention and sexual coercion can be legally addressed, the third category, gender harassment, has been recognized as being the least acknowledged yet most pervasive form of sexual harassment (Fairchild et al., 2018). Gender harassment does not aim at sexual favours or coercion. Rather, it aims at putting down and pushing out individuals who do not conform to the individualistic and competitive norms of the workplace, by hostile attitudes and derogating, demeaning, humiliating, and denigrating behaviours (Berdahl, 2007). Gender harassment is an expression of power and dominance, and as such well-suited to protect and enhance individual status in an existing gender hierarchy (Berdahl, 2007). The Iceberg model of sexual harassment (NASEM, 2018; Cortina and Areguin, 2021) clearly shows that the bulk of sexual harassment are ‘put downs’ (gender harassment). However, these are typically below the surface, while the comparatively rare ‘come ons’ (unwanted sexual attention and sexual coercion) are often more high-profile and media-prone.

In spite of universities’ commitments to being inclusive, safe, and equitable working environments, the ineffectiveness of anti-harassment and non-discrimination policies in higher education has been demonstrated by numerous reports over the past years. Two reports in Netherlands show that harassment in Dutch academia is pervasive (Naezer et al., 2019; Young Academy Groningen Report, 2021). Both reports point to pervasive experiences by female scholars of their career being sabotaged and obstructed. Examples for this are vague and changing performance criteria used to deny promotion, being excluded from opportunities to professionalize or to develop relevant skills and being denied tenure. In addition, the report by the Young Academy Groningen Report (2021; see also Hilton and Täuber, 2022) points to the high prevalence of intersecting disadvantages among interviewees. Of the self-selected sample of 26 current and former members of staff, 22 were women and four men, 23 were international and three were Dutch. Importantly, all Dutch participants were female, and all internationals participants were male. One scholar put their experiences like this: “It’s the first time in my life that I think so strongly that I am a young woman in a university and also a migrant. And I have lived and worked in many other countries outside of the country where I was born. … I never had so many times in my life that I’m called international. I was always a colleague, nobody referred to me as an international woman.” In the described context, “international” referred to anyone of non-Dutch origin. The qualitative approaches taken in the Dutch reports are complemented by a quantitative online survey on academic working culture in the United Kingdom, conducted by the Wellcome Trust (2020) among over 4,000 researchers. The survey showed that many respondents experienced bullying, discrimination, harassment, and exploitation, leading to a sense of isolation and loneliness, mental health problems, and anxiety. Importantly, here, too, researchers from underrepresented groups faced the most challenges.

These reports suggest that intersectional approaches would be of added value for examining academia as an organizational working environment. This aligns with observations that women belonging to several disadvantaged identities face more harassment (e.g., Oertelt-Prigione, 2020; Moss and Mahmoudi, 2021), whereas interventions to promote women in academia typically only cater to a narrowly defined range of white, cis-gender, straight, middle-class women, and therefore, often fail to realize the intended benefits for less privileged women (e.g., Täuber, 2019; Tzanakou, 2019). However, while intersectionality could be a useful policy tool for academia as an institution, it has not been widely used to examine inequality production and reproduction in academia (Jouwe, 2015). Especially in quantitative research, intersectional approaches are still relatively uncommon (NASEM, 2018; Bondestam and Lundqvist, 2020; Cortina and Areguin, 2021).

### Intersectionality

As outlined in the introduction, European higher education policy largely ignores intersectional approaches. When intersectionality is considered at all, it often refers to weak intersectionality and additive approaches (Bowleg, 2008; Grzanka, 2018; Bauer et al., 2021), which differ from intersectional analysis rooted in Black feminist movements (Crenshaw, 1989, 1991; Wekker, 2016). Statements about the lack of an intersectional approach to anti-harassment and non-discrimination policy in academia (Bondestam and Lundqvist, 2020) point to policies that indiscriminately focus on women, neglecting the heterogeneity of “women scholars” (Atewologun and Sealy, 2014; Atewologun et al., 2016). Śliwa and Johansson (2014) examined the career trajectories of foreign women—a term respondents used to self-describe “a relational, heterogeneous category of workers, for whom depending on the situation and the individual, career trajectories will be influenced by different factors, not always affecting exclusively foreign women” (p. 829)—and found that the career trajectories and progressions of that group were affected by multiple bases of organisational inequalities. These inequalities could be based on gender, on affiliation with a particular nationality or religion, but also on accent.^1^ Similarly, members of minoritized groups in organizations are disproportionately confronted with sexual harassment (Healy et al., 2019; Oertelt-Prigione, 2020), discrimination (Young Academy Groningen Report, 2021), and bullying (Moss and Mahmoudi, 2021). Clancy et al. (2017), for instance, show that in astronomy and planetary science, women of colour face greater risks of gendered and racial harassment.

Prior research has criticized the lack of studies that consider how inequalities related to gender, race, and class mutually reinforce or contradict each other (Acker, 2006). Intersectional approaches can help to disentangle and make visible interacting systems of inequality (Boogaard and Roggeband, 2010), but might be constrained by pragmatic, legislative, or ideologic considerations. In such cases, quantitative approaches, although being suboptimal by engaging weak and additive approaches to intersectionality (Bowleg, 2008; Grzanka, 2018; Bauer et al., 2021), might fulfil a signalling function for areas requiring follow-up research employing better suited approaches. Arguably, in academic contexts, where career progress is typically contingent on scholars’ visibility, ignoring intersectional disadvantages will contribute to policy-practice gaps. Importantly, intersectionality also creates specific privileges. In an academic context, Miller and Roksa (2019) showed how combined racial and gender privilege places white men in the most advantaged, and racial/ethnic minority women in the most disadvantaged, position in terms of protected research time, opportunities for collaborations, and building networks. Similarly, research in a Dutch business school demonstrated higher salary and rank of Dutch men compared to non-Dutch women (Bago D’Uva and Garcia-Gomez, 2020). The intersectional aspect is under-researched but important to consider because it can be both a cause and a consequence of policy ineffectiveness (Moughalian and Täuber, 2020), especially because it might be associated with psychological safety and voice.

### Psychological Safety

Psychological safety denotes individuals’ positive assumptions about how the other party might react when asking something or reporting a problem. Roussin and Webber (2011), for instance, define psychologically safe working environments as high in trust, encouraging risk-taking and thus vulnerability, such that employees do not need to be concerned about their jobs or reputation. Thus, experiencing an open environment, for instance, contributes to feelings of psychologically safety (Edmondson, 2003). On the other hand, employees feel abandoned in organizations that fail to ‘walk the talk’. This is due to a perceived violation of the psychological contract between the employee and the organization (Cartwright and Cooper, 1996; Morrison and Robinson, 1997). For instance, when harassment is frequent despite anti-harassment policies, employees’ expectation that the institution protects them is violated. In order to feel psychologically safe, employees need to trust their institution, which is more difficult when decoupling indicates word-deed discrepancies (Zhang et al., 2010). Being mistreated and discriminated against can harm the target’s psychological safety (e.g., Castaneda et al., 2015).

### Voice

Women scholars’ willingness to voice their experiences is important, not only for them individually to claim their space, but also for organizations to learn about possible discrepancies between policy and practice. Gender equality interventions, for instance, are typically designed with white middle-class women in mind (e.g., Clavero and Galligan, 2021) and often neglect the specific disadvantages faced by women scholars with intersecting disadvantages. If these scholars are not feeling safe to share their experiences and how the interventions let them down, improvement of such programs and interventions is unlikely. Testifying to this, a feminist disability rights activist states (MamaCash, 2022), “if you stay quiet, you stay invisible.”

Liang et al. (2012) refer to employees’ voice as raising ideas and speaking up about problems. In addition to attitudes, McLaughlin et al. (2012) point out that women experiencing harassment are more likely to leave their career and look for different jobs elsewhere. Fear of retaliation is a strong inhibitive force to voicing experiences of mistreatment (e.g., Cortina and Magley, 2003). Institutions are often permissive of retaliation against reporters of harassment because retaliation helps to retain hierarchical power systems (Near et al., 1993; Svensson and Genugten, 2013). After all, harassment is typically intended to dominate and assert control over the target (McLaughlin et al., 2012; Medeiros and Griffith, 2019). When voicing such experiences, for instance through reporting them, targets of harassment undermine the mechanism intended to submit them. Because strongly hierarchical organizations such as academia (NASEM, 2018) endow power and privilege to those at the top of the hierarchy, typically majority members, reporting harassment will often be seen as a threat to the hierarchy.

Supporting this proposition, institutions—including academia—have been found to engage in various ways to silence targets of harassment. Fernando and Prasad (2018), for instance, show that various organizational actors such as HR, line managers, and colleagues, mobilize various discourses to persuade reporters not to voice their discontent. Ultimately, these authors find that many targets of harassment at universities are tricked into ‘reluctant acquiescence’ and self-silence. As a result, targets of harassment can feel betrayed by the institution, which often is related to complex trauma and damage to mental health (Harsey et al., 2017). Even without the involvement of additional organizational actors besides the perpetrator, not speaking up about unfair treatment and harassment can negatively affect employees’ mental health (Cortina and Magley, 2003). Thus, voicing experiences of mistreatment is important for individual mental health as well as for signalling to organizations that change is necessary.

#### Intersectionality, Psychological Safety, and Voice

Intersectional approaches to psychological safety seem warranted given that members of sexual minority groups often are exposed to more harassment and feel less psychologically safe (Silverschanz et al., 2007). Feeling psychologically safe is essential for voicing experiences of mistreatment (Walumbwa and Schaubroeck, 2009; Singh et al., 2013). Indeed, employees who feel psychologically safe are more likely to report institutional misconduct such as sexual harassment, unfair treatment or other unethical incidents (Walker et al., 2019; Edmondson, 2020). Intersectional approaches are relevant for voice, too. Women of colour are often dismissed as the “angry black women” because they are seen as masculine and aggressive, which can undermine their willingness to voice experiences of harassment in an attempt to not confirm these stereotypes (Hall et al., 2019). When it comes to harassment in the workplace, women belonging to minority groups are targeted more than women belonging to the majority (Berdahl and Moore, 2006). In an attempt to complement the “put downs” associated with silencing women scholars with the “push outs,” women scholars’ career choices will be accounted for in the survey, too. Thus, policy ineffectiveness is expected to relate negatively to women academics’ willingness to be vocal about their experience because it undermines their feelings of psychological safety.

## The Current Study

**Figure 1:**
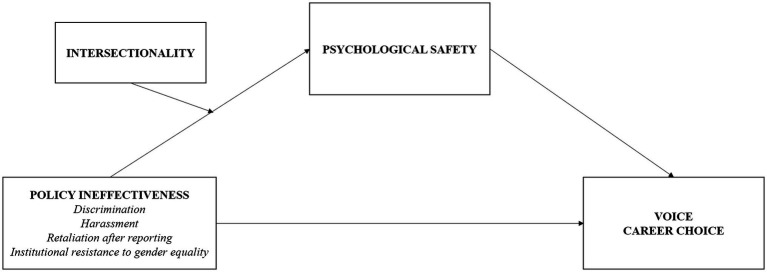
Conceptual model tested in this research.

Given that especially quantitative research into sexual harassment is sparse (Healy et al., 2019; Cortina and Areguin, 2021), the present paper investigates how policy ineffectiveness—operationalized as experiences of harassment, discrimination, retaliation for reporting, and institutional resistance to gender equality—relates to women academics’ feelings of psychological safety, voice and career choices, and whether these relationships are more pronounced for women facing intersectional disadvantage. The following hypotheses were tested: Policy ineffectiveness, operationalized as harassment, discrimination, retaliation after reporting, and institutional resistance, is expected to be negatively associated with feelings of psychological safety (*Hypothesis 1*). Policy ineffectiveness is expected to be negatively associated with voice and career choices favouring academia (*Hypothesis 2*). Psychological safety is expected to be positively associated with voice and career choices favouring academia (*Hypothesis 3*). Intersectionality is expected to strengthen the negative association between policy ineffectiveness and psychological safety (*Hypothesis 4*). Thus, the complete conceptual model (see Figure 1) to be tested predicts a moderated mediation, with intersectionality and policy ineffectiveness interactively affecting voice and career choice through psychological safety.

### Methods

#### Power Analysis

The preregistration of the study in the Open Science Framework (OSF) explains how the number of required participants was calculated.^2^ *A priori* power analysis conducted with G*Power Faul et al., 2009) revealed that a minimum of 54 participants were required, based on the three predictors of policy ineffectiveness, intersectionality, and psychological safety to test the hypotheses. The aim was to recruit as many participants as possible within a timeframe of 6 weeks, but a minimum of 54. Women from various international universities were contacted, including women from the Netherlands, Germany, Austria, and other countries through the author’s professional network. Potential participants were contacted *via* email and were asked to participate in the survey. Before filling out the survey, participants filled in an informed consent form that was part of the survey.

#### Participants and Procedure

Potential respondents were invited through the author’s professional networks, who sent an email explaining the goal of the research, inviting the addressee to participate, and provided a link to the Qualtrics survey. The research was introduced as an attempt to study academics experiences with gender inequality. Addressees were encouraged to share the survey link with other potentially interested people (snowballing). Before being presented with the survey questions, participants gave informed consent. Two-hundred-and-four people started the survey, but only 100 filled it out completely, of whom 91 were women. These were the 91 respondents included in the analyses presented below. Respondents worked at their current institution on average for 8.29 years (*SD* = 6.97). Forty-two respondents were tenured, 46 were not tenured, and three did not wish to disclose that information (46.2, 50.5, and 3.3%, respectively). Respondents had 28 different nationalities, with most from the Netherlands (28.7%), followed by Austria (13.2%), and Germany (12.1%). Respondents reported 20 different current countries of residence, most in the Netherlands (47.3%), Austria (14.3%), and the United Kingdom and Northern Ireland (6.6%). At the time of the survey, 43 respondents (52.7%) worked in a different country than their country of origin. No difference was observed in frequency of tenure as a function of whether a women scholar was foreign (tenured: *N* = 20, not tenured: *N* = 22) or domestic (tenured: *N* = 22, not tenured: *N* = 24), *χ*^2^(2) = 0.05, *p* > 0.83. Respondents worked in various universities and scientific fields, with most of them from Social and Behavioral Sciences (45.1%), followed by Business and Economics (22%), and Arts and Humanities (13.2%).

#### Measures

Unless indicated otherwise, all items were measured on 5-point Likert scales ranging from 1 (*not at all*/*completely disagree*) to 5 (*very much*/*completely agree*). The complete questionnaire can be found in the Appendix A.

In order to create an additive index of *intersectionality*, respondents were asked to indicate whether they differed from the majority of the people they work with at their current institution for a range of factors adopted from the Athena Survey of Science, Engineering and Technology (2016). These factors included, among others, race, ethnicity, sexual orientation, disability status, and religious affiliation. The perceived effects of these factors on respondents’ careers were assessed with the question “Please rate the extent to which these dimensions have affected your career and career choices to date,” ranging from *extremely negative* to *extremely positive*. Complementing questions about disadvantage, respondents’ understanding of the attributes that are associated with *privilege in resource allocation* was assessed with the question “In your academic environment, what kind of attributes would a person need to have in order to be most favoured/privileged in resource allocation?.” Respondents could provide their answers using eight open answer text boxes.

*Policy ineffectiveness* was measured with the four constructs that indicate ineffectiveness of non-discrimination and anti-harassment policies, inadequacy of complaint management procedures, and lack of success in implementing gender equality interventions. These constructs were discrimination, harassment, retaliation after reporting, and institutional resistance to gender equality. In order to facilitate a shared understanding of the terms, definitions were provided before presenting the items. *Discrimination* was measured with 15 items (*α* = 0.90) adapted from the Athena Survey of Science, Engineering and Technology (2016). Respondents were asked to indicate “In your main academic working environment, how common is resource allocation that favors men or other academics that are more similar to the majority group?,” where low values reflect that discriminatory resource allocation is very uncommon, and higher values reflect that it is very common. Thus, different from traditional discrimination scales, these items did not ask for individual experiences of discrimination *per se*, but more for how normalized and common discrimination is in respondents’ academic working environment. *Harassment* was measured with 6 items (*α* = 0.90) adapted from different sources (Naezer et al., 2019; Cortina and Areguin, 2021). *Retaliation after reporting* was measured with 12 items (*α* = 0.95) adapted from Svensson and Genugten (2013) to match the context of gender equality. This scale was preceded by the question “Have you ever complained about harassment or discrimination, or do you know of other who have done so?.” *Institutional resistance to gender equality* was measured with 8 items (*α* = 0.87) that were developed based on a recently developed model of institutional resistance towards initiatives to advance gender equality, ranging from passive to active forms of resistance (Flood et al., 2021).

*Psychological safety* was measured with seven items (*α* = 0.83) adapted from Edmondson (1999). Higher values on this scale indicate that participants feel more psychologically safe in their institutions.

*Voice* was measured with 10 items (*α* = 0.92) adapted from Liang et al. (2012) to match the context of gender equality (e.g., “I proactively develop and make suggestions for issues that may influence gender equality in my working environment”; “I dare to voice out opinions on gender inequality, even if that would embarrass others”). Higher values on this scale mean that respondents feel more at ease to letting themselves be heard.

*Career choices* were measured with four self-developed items (e.g., “I consider changing careers,” reverse coded; *α* = 0.80). Higher values on this scale indicate that respondents would like to continue their career in academia and would recommend this to other women, too.

### Results

#### Intersectionality

**Figure 2:**
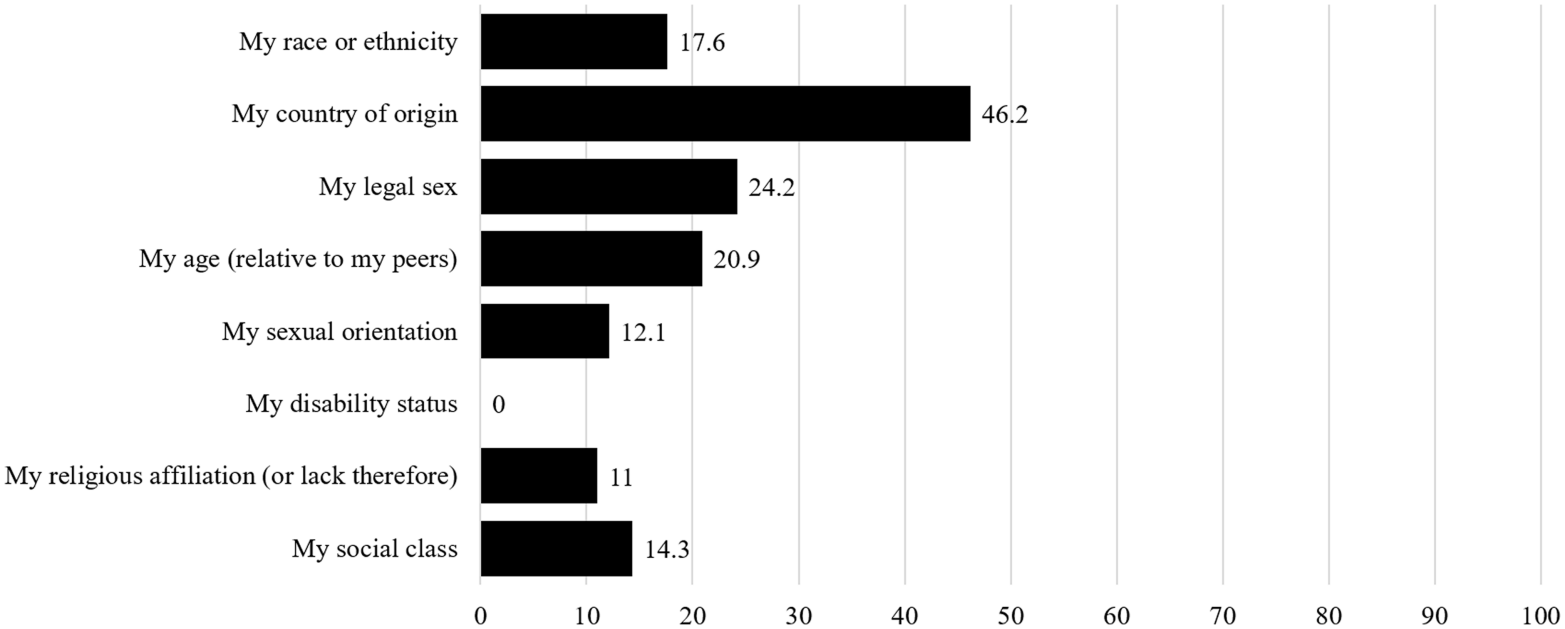
Respondents’ answers to the question “Regarding which attributes do you differ from the majority at your workplace?”

Figure 2 provides an overview over the percentages with which the different dimensions were named. Respondents indicated not differing at all from the majority of people they worked with (23.1%), differing on one dimension (35.2%), on two (18.7%), on three (12.1%), four (7.7.%) and five dimensions (3.3%). On average, respondents indicated differing from the majority at their current university on 1.56 dimensions (*SD* = 1.35). For the correlation and moderation analyses, intersectionality has been calculated as a sum, with 0 indicating no difference from the majority, and 5 indicating differing from the majority on five dimensions.

**Figure 3:**
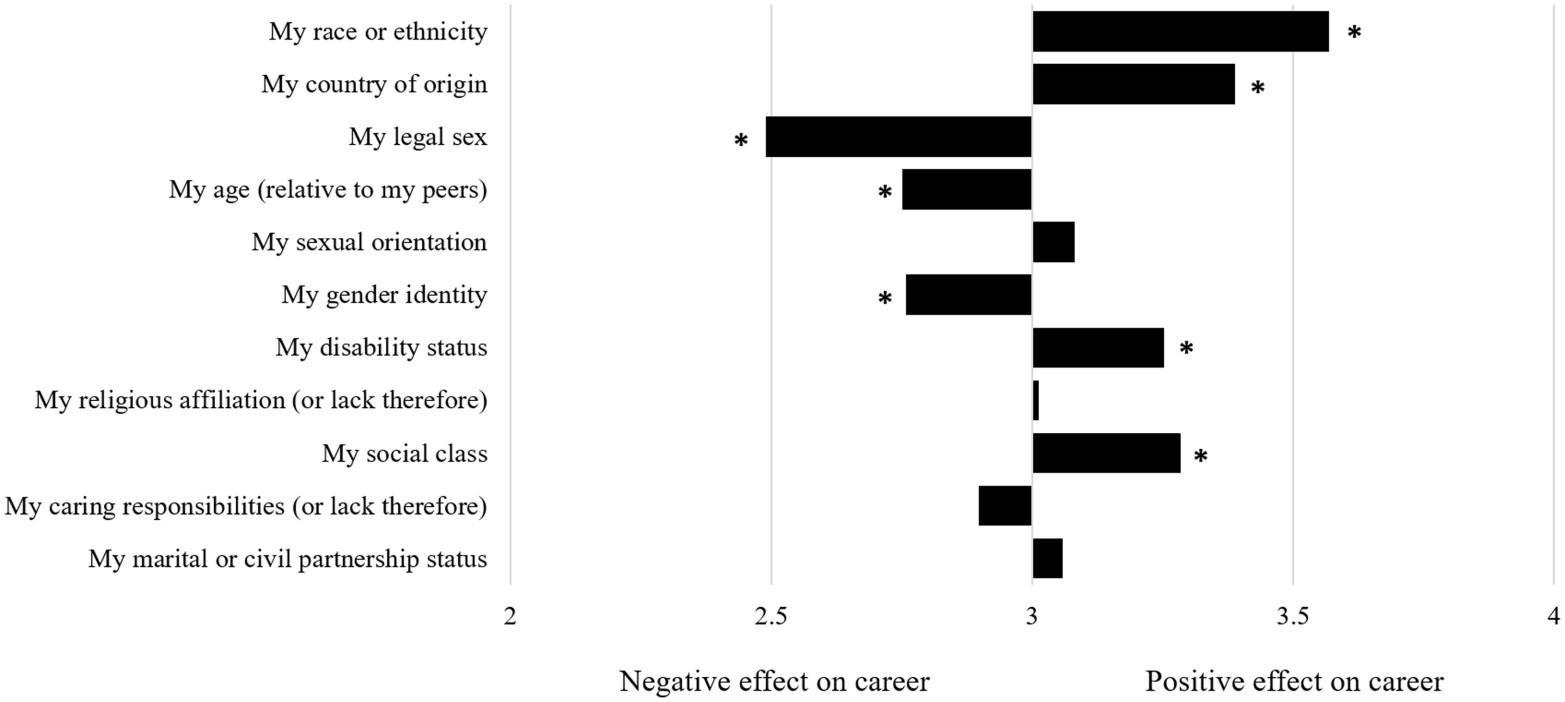
Respondents’ answers to the question “Please rate the extent to which these dimensions have affected your career and career choices to date.” Asterisks (*) denote significant differences from the scale mean.

Examining respondents’ understanding of the impact of different factors on their career using a one-sample *t*-test shows that they are aware of privileges they have, too (see Figure 3). For instance, the predominantly white sample indicates that their race/ethnicity is an advantage for their career [*M* = 3.57, *SD* = 0.93, *t*(87) = 5.72, *p* < 0.001], as are their countries of origin [*M* = 3.39, *SD* = 1.01, *t*(89) = 3.64, *p* < 0.001], which are mostly Western, their social class [*M* = 3.28, *SD* = 0.98, *t*(87) = 2.71, *p* = 0.008], and the fact that they are able-bodied [*M* = 3.25, *SD* = 0.77, *t*(86) = 3.08, *p* = 0.003]. At the same time, sex (*M* = 2.49, *SD* = 0.89), age (*M* = 2.75, *SD* = 0.76), and gender identity (*M* = 2.76, *SD* = 0.79) were factors perceived as exerting significantly negative effects on respondents’ academic careers (all *t*’s > −2.80, all *p*’s < 0.007). Factors not associated with significant disadvantage or privilege were sexual orientation, religious affiliation, caring responsibilities, and marital or civil status.

#### Descriptive Analyses

Table 1 provides an overview over means, standard deviations, and correlations of the constructs of interest. Means of constructs related to *Policy ineffectiveness* were all significantly above the mid-point of the respective scale, all *t*’s > 5.00, all *p*’s < 0.001. This suggests that women academics taking part in this research were rather familiar with discrimination, harassment, and institutional resistance to gender equality. Respondents were also familiar with retaliation after reporting harassment, which is concerning given that more than two-thirds of the study’s respondents indicated having complained about harassment or discrimination themselves (*n* = 31), or knowing of others who have complained (*n* = 55). Only 25 respondents indicated not having complained themselves and not knowing others who have done so.

**Table 1:** Means, standard deviations, and correlation coefficients.

	1	2	3	4	5	6	7	8
1. Intersectionality	1.57 (1.35)							
2. Discrimination	0.35**	3.66 (0.63)						
3. Harassment	0.24**	0.44**	3.03 (1.00)					
4. Retaliation	0.30**	0.52**	0.72**	3.23 (0.95)				
5. Resistance	0.30**	0.54**	0.59**	0.61**	3.48 (0.82)			
6. Psychological Safety	−0.28**	−0.36**	−0.60**	−0.58**	−0.46**	3.20 (0.85)		
7. Voice	0.20	0.32**	0.32**	0.27**	0.43**	−0.09	3.63 (0.81)	
8. Career Choice	−0.19	−0.31**	−0.24*	−0.35**	−0.30**	0.40**	−0.15	3.18 (1.05)

^*^p < 0.05, ^**^p < 0.01. Means and standard deviations are provided in the diagonal.

The correlation analysis suggests that intersectionality is associated with more discrimination, harassment, retaliation after reporting discrimination and harassment, and institutional resistance to gender equality. Intersectionality also relates to lower reported psychological safety. Regarding voice, women academics indicate speaking up more when confronted with higher levels of discrimination, harassment, retaliation after reporting and institutional resistance. However, when career choices are concerned, higher levels of discrimination, harassment, retaliation after reporting and institutional resistance are associated with stronger considerations to change institutions and career.

#### Moderation Analysis

Based on the correlation patterns, moderation analyses were performed to test whether policy ineffectiveness is more strongly negatively related to psychological safety for women academics who indicated differing from the majority on multiple dimensions. The analyses reveal that in particular experiences of institutional resistance to gender equality are related to psychological safety more negatively for women academics who differ from the majority on more dimensions (Table 2), whilst the interaction is marginally significant for experiences of harassment. The patterns for experiences of discrimination and retaliation after reporting show the same direction, but are not significant. The simple slopes analyses presented in Table 3 show that the associations between policy ineffectiveness and psychological safety are generally stronger and more negative for women academics with more (see column on the right) compared to less intersecting disadvantages.

**Table 2:** Moderation of the association between resistance-related experiences and psychological safety by intersectionality.

Dependent variable: Psychological safety					
Independent variables	Effect (SE)	*t*	LLCI	ULCI	Model summary
**Discrimination**	−0.29 (0.10)	−2.84^*^	−0.5004	−0.0885	Total *F*(3,87) = 6.38, *p* < 0.001, *R^2^* = 18.03% Interaction *F*(1,87) = 2.34, *p* = 0.130
Intersectionality	−0.15 (0.10)	−1.39	−0.3540	0.0627	
Interaction	−0.15 (0.06)	−1.53	−0.3363	0.0437	
**Harassment**	−0.57 (0.09)	−6.63^**^	−0.7380	−0.3977	Total *F*(3,87) = 6.38, *p* < 0.001, *R^2^* = 40.11% Interaction *F*(1,87) = 3.77, *p* = 0.056
Intersectionality	−0.08 (0.09)	−0.86	−0.2598	0.1022	
Interaction	−0.17 (0.09)	−1.94^‡^	−0.3495	0.0042	
**Retaliation**	−0.59 (0.09)	−6.49^**^	−0.7730	−0.4105	Total *F*(3,87) = 17.62, *p* < 0.001, *R^2^* = 37.79% Interaction *F*(1,87) = 2.85, *p* = 0.095
Intersectionality	−0.06 (0.09)	−0.69	−0.2492	0.1212	
Interaction	−0.17 (0.09)	−1.69	−0.3613	0.0293	
**Resistance**	−0.40 (0.10)	−4.23^**^	−0.5917	−0.2135	Total *F*(3,87) = 11.46, *p* < 0.001, *R^2^* = 28.33% Interaction *F*(1,87) = 5.86, *p* = 0.018
Intersectionality	−0.07 (0.10)	−0.65	−0.2680	0.1366	
Interaction	−0.26 (0.11)	−2.42^‡‡^	−0.4819	−0.0474	

^‡^p = 0.056, ^‡‡^p = 0.018, ^*^p < 0.01, ^**^p < 0.001.

**Table 3:** Simple slopes analysis for different levels of intersectionality.

	Low intersectionality (−1 SD)		High intersectionality (+1 SD)	
	*ß*	*t*	*ß*	*t*
Discrimination	−0.15	−1.02	−0.44	−3.23^**^
Harassment	−0.40	−3.25^**^	−0.74	−5.92^***^
Retaliation	−0.43	−3.64^***^	−0.76	−5.08^***^
Resistance	−0.14	−0.93	−0.67	−4.75^***^

^**^p < 0.01, ^***^p < 0.001.

#### Moderated Mediation Analysis

Moderated mediation analysis tests the conditional indirect effect of a moderating variable on the relationship between a predictor and an outcome variable *via* a mediator variable. The correlation matrix in Table 1 shows that psychological safety is associated with career choices, but not with voice. The moderation analysis above shows that the association of institutional resistance with psychological safety is significantly, and the association of harassment with psychological safety is marginally moderated by intersectionality. Consequently, the moderated mediation hypothesis was tested with two separate models with harassment and institutional resistance as independent variables, respectively, psychological safety as mediator, and intersectionality as moderator. In both models, career choice is the dependent variable. Using PROCESS model 7 (Hayes, 2018; 5,000 bootstrap samples, predefined), this revealed that higher levels of *harassment* were related to lower levels of psychological safety (Table 4, left panel, mediator model). Higher levels of psychological safety, in turn, were associated with career choices in favour of academia (Table 4, left panel, dependent model). The conditional indirect effect of *harassment* on career choice *via* psychological safety was significant at all levels of the moderator, but was strongest at high, and weakest at low levels of intersectionality (Table 5, left panel). The overall moderated mediation model was supported by a reliable index of moderated mediation (−0.07) as indicated by zero not being included in the confidence interval (*CI_95%_* = −0.1741; −0.0021).

**Table 4:** Moderated mediation results for harassment (left panel) and resistance (right panel).

	Mediator model				Mediator model		
	DV=Psych. Safety, *R^2^ =* 40.11%				DV=Psych. Safety, *R*^2^ = 28.33%		
Predictor	*b*	*SE*	*t*		*b*	*SE*	*t*
Constant	0.04	0.09	0.40	Constant	0.07	0.10	0.80
Harassment	−0.57	0.09	−6.63^***^	Resistance	−0.40	0.10	−4.23^***^
Intersectionality	−0.08	0.09	−0.87	Intersectionality	−0.07	0.10	−0.65
Interaction	−0.17	0.09	−1.94^‡^	Interaction	−0.26	0.11	−2.42^*^
	**Dependent variable model**				**Dependent variable model**		
	**DV=Career Choice, *R^2^ =* 15.97%**				**DV=Career Choice, *R^2^ =* 17.58%**		
Constant	0.00	0.10	0.00	Constant	0.00	0.10	0.00
Harassment	−0.00	0.12	−0.03	Resistance	−0.14	0.11	−1.31
Psych. Safety	−0.40	0.12	3.26^**^	Psych. Safety	0.33	0.11	3.06^*^

^‡^p = 0.056, ^*^p < 0.05, ^**^p < 0.01, ^***^p < 0.001.

**Table 5:** Moderated mediation results examining conditional indirect effects of harassment (left panel) and resistance (right panel) on career choice *via* psychological safety at different levels of the moderator intersectionality.

	Harassment				Resistance			
Intersectionality	Effect	BootSE	LLCI	ULCI	Effect	BootSE	LLCI	ULCI
−1.16	−0.15	0.08	−0.3246	−0.0250	−0.03	0.07	−0.1889	0.0749
−0.42	−0.20	0.08	−0.3847	−0.0575	−0.10	0.06	−0.2369	−0.0191
1.06	−0.30	0.13	−0.5784	−0.0834	−0.23	0.08	−0.4177	−0.0848

Similarly, higher levels of *institutional resistance* were related to lower levels of psychological safety (Table 4, right panel, mediator model). Higher levels of psychological safety, in turn, were associated with career choices in favour of academia (Table 4, right panel, dependent variable model). The conditional indirect effect of *institutional resistance* on career choice *via* psychological safety was strongest and significant at high levels of intersectionality, weaker but still significant at medium levels, and was non-significant at low levels of intersectionality (Table 5, right panel). The overall moderated mediation model was supported by a reliable index of moderated mediation (−0.09) as indicated by zero not being included in the confidence interval (*CI*_95%_ = −0.1831; −0.0132). Together, the analyses suggest that particularly for women academics who differ from the majority on multiple dimensions, experiences of harassment and institutional resistance undermine their feelings of psychological safety, making them consider to leave their institution or their career more strongly.

#### Who Enjoys Privilege in the University?

**Figure 4:**
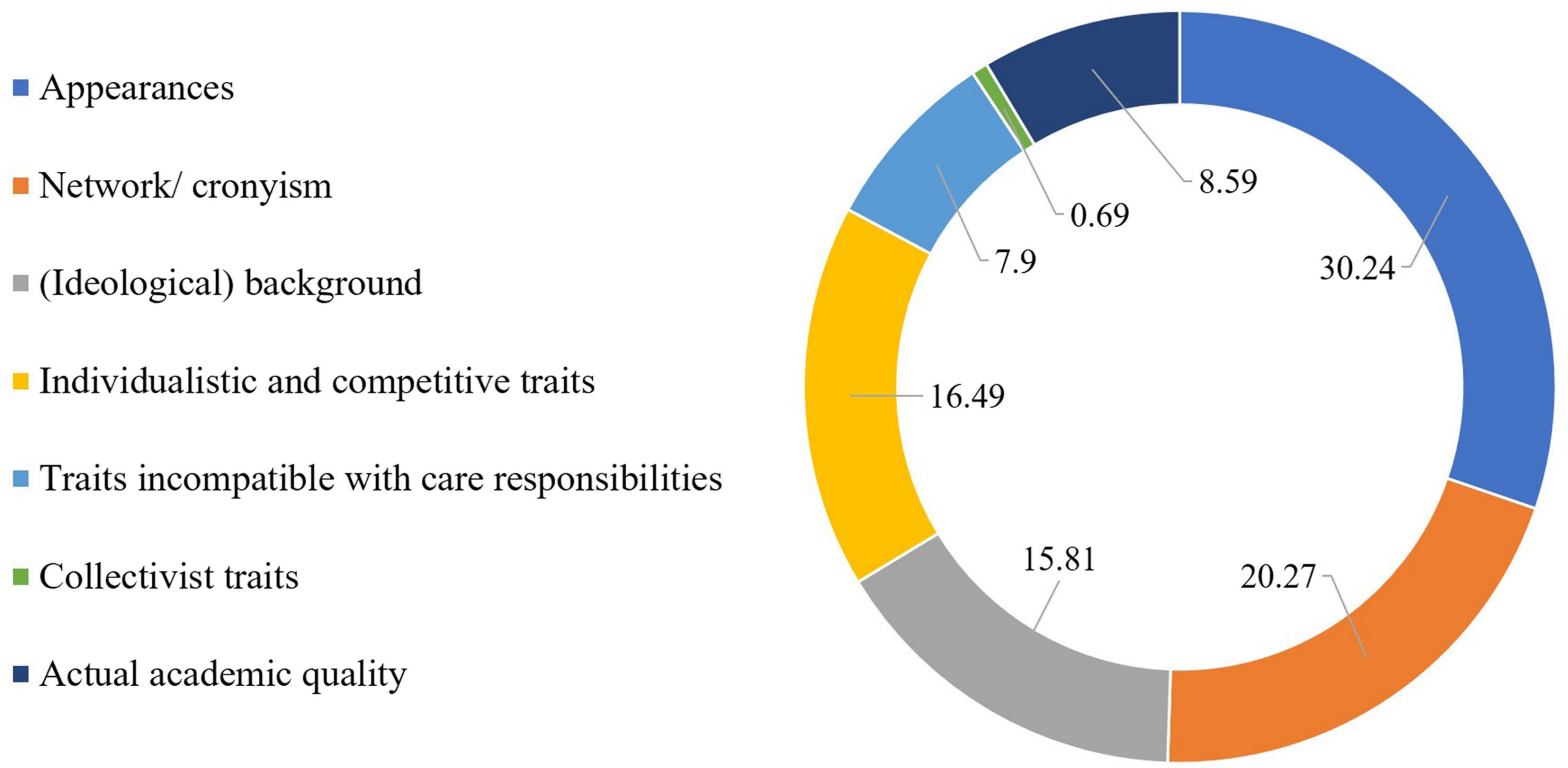
Attributes affording privilege in academia.

Respondents were asked to provide up to eight attributes that a person would need to have in order to be most favoured or privileged in respondents’ academic environment. They produced almost 300 attributes which were summarized in thematically coherent clusters by the author and a research assistant. We each clustered 30 attributes and compared the theme we assigned to each attribute. Differences in clustering decisions were discussed and resolved. Overlapping themes were labelled consistently. Table 6 provides an overview of the subtopics clustered in overarching themes. The generated attributes reflect the privilege that arises from belonging to majority on multiple dimensions. Figure 4 shows that the most privileged individuals in respondents’ academic environment are white, middle-aged, heterosexual males who display characteristics that are typically associated with individualism and competitiveness, such as being assertive, outspoken, and self-assured. Many respondents refer to networking skills and the willingness and ability to connect with powerful others, which is sometimes referred to as cronyism. A topic cross-cutting the thematic clusters is being embedded in local, close-knitted strategic and political networks, which seem instrumental for getting ahead. Only two codes referred to traits that are associated more strongly with collectivism, namely being a team player and caring. Freedom from care responsibilities was a theme in and of itself, as it enables academics to be always available, working fulltime, and be flexible, and hence to be more productive and visible. Academia as a system thus is seen as favouring people based on traits that hardly relate to actual academic merit. Indeed, qualities such as education or being an expert were named only 25 times (8.59%) as a reason for enjoying privilege in the university. However, 13 of those attributes are known to be heavily gendered, such as number of publications, external recognition and the ability to attract funding (e.g., Clavero and Galligan, 2021). In sum, of the entire range of attributes that respondents associate with being privileged in the academy, only about 4% were related to actual academic skills.

**Table 6:** Thematic clustering of attributes applying to those who are privileged in the academic environment, as generated by female scholars.

Factors	Count	% of factor	% of total
**Appearances**	**88**		**30.24**
Male	46	52.27	
White	26	29.55	
Seniority/age	12	13.64	
Able-bodied	2	2.27	
Slim	1	1.14	
Taller than average	1	1.14	
**Network/cronyism**	**59**		**20.27**
Political and local connections/cronyism	30	50.85	
General network skills	10	16.95	
Being seen by those in power	10	16.95	
Conforming with those in power/the majority	9	15.25	
**(Ideological) background**	**46**		**15.81**
Local nationality/Western	17	36.96	
Speaks native language	10	21.74	
Heteronormative	8	17.39	
Middle or upper class	7	15.22	
Conservative	3	6.52	
Neurotypical	1	2.17	
**Individualistic and competitive traits**	**48**		**16.49**
Outspoken	13	27.08	
Assertive	12	25.00	
Confident	12	25.00	
Career-Minded	11	22.92	
**Collectivist traits**	**2**	100.00	**0.69**
**Traits less compatible with caring responsibilities**	**23**	100.00	**7.90**
**Actual academic quality**	**25**		**8.59**
Competence/qualification	13	52.00	
Publications	7	28.00	
Funding	5	20.00	
	291		100

Bold values indicate overarching themes.

## Discussion

Policy ineffectiveness, operationalized as experiences of harassment, discrimination, retaliation for reporting misconduct, and institutional resistance against gender equality, is associated with lower levels of psychological safety and undermines women scholars’ willingness to stay in their working environment. The negative association between policy ineffectiveness and psychological safety was stronger among women academics who indicated that they differed from the majority in their institution on multiple dimensions. Support for the moderated mediation hypothesis was found in particular for institutional resistance to gender equality, and marginally for harassment. Both components were more strongly negatively associated with psychological safety among women academics facing intersectional disadvantages. Psychological safety was positively associated with career choices in favour of academia, meaning that women who felt psychologically safe were more likely to recommend working in academia to other women and their daughters and were less inclined to leave their university or academia altogether. The negative effects of harassment and institutional resistance on career choices were fully mediated by perceived psychological safety. The study thus shows that ineffective policy is not just disappointing on an institutional level, but that it contributes to the reproduction of a rather homogenous academic community that does not do justice to the wealth of perspectives that women academics with diverse social positions can offer.

The approach to intersectionality explored here is conscious of its shortcomings and benefits. Shortcomings involve the weak conceptualization of intersectionality that is inherent to an additive approach to multiple identities as used here (Bowleg, 2008; Grzanka, 2018) and to quantitative research endeavours more generally (Bauer et al., 2021). Benefits pertain to demonstrated usefulness of the employed self-report measurements to signal intersectional disadvantage as well as awareness of own and others privilege, thereby creating valuable insights despite pragmatic, legislative, or ideologic constraints to studying intersectionality. Thus, the approach used here can be instrumental for pointing towards areas that require more in-depth research attention in order to design effective policy and implement it successfully. This applies to intersectional disadvantages experienced by women scholars, as well as to relationality (i.e., in comparison with whom am I privileged or disadvantaged?) and social constructionism (i.e., which traits and characteristics “make” an academic; Windsong, 2018). Participants were aware of their positionality in terms of benefitting from versus being disadvantaged in their respective academic working environment (see Figure 3).

I thus conclude that the approach that I have used here can be useful to combine the desire for intersectional approaches with reality constraints that might apply. In the European context, such constraints pertain primarily to very strict privacy laws (Goddard, 2017) and to the depoliticization of race and how it relates to power (Rose, 2022). In order to understand how oppression and privilege contribute to the perpetuation and reproduction of inequality in academia, and thus to design and implement effective policy, the approach I have used here needs to be follow-up by inquiries into strong intersectionality (Bowleg, 2008; Grzanka, 2018; Windsong, 2018). This is essential for optimally using intersectionality as a critical framework to understand “the ways in which heterogeneous members of specific groups (such as women) might experience the workplace differently depending on their ethnicity, sexual orientation, and/or class and other social locations” (Atewologun, 2018, p. 1). But how can such follow-up research be stimulated?

Change initiatives are often mandated to those in powerful positions. Particularly where policy aims to further progressive change, such as making academia a more inclusive, equitable, and safe working environment, the desire to maintain power and privilege might undermine effective policy implementation. Powerful groups are notoriously known for their opposition to change that might threaten their privileged position (Dixon et al., 2010; Blader and Chen, 2011; Marr and Thau, 2014; Dover et al., 2016). Unfortunately, the lack of strong approaches to intersectionality means that discourses of power, privilege, and system-supporting inaction are also missing in analyses of policy-practice gaps. The weak approach to intersectionality in higher education policy might therefore be an example of power exertion through discourse (Lukes, 2004; Cath et al., 2014; Rose, 2022) and co-optation (Gaventa, 2006). The term ‘intersectionality’ might then be used to suggest analytical and philosophical engagement with systemic injustice, but can neither address the co-constitution of multiple identities, nor contribute to meaningful analyses of power and privilege or social constructivism (Atewologun, 2018). Indeed, Cath et al. (2014) observed that the Dutch culture is both colour-blind and power-blind, and the same can likely be said for other academic cultures.

That considerations of power and privilege need to be included in the design, and implementation of policy in higher education was clear from women scholars’ ability to clearly pinpoint intersectional privilege. Respondents produced a very clear prototype of who is enjoying privilege and power in the academic environment. This prototype combined a set of attributes that were not at all meritocratic, but rather arbitrary in relation to academic qualities and skills. The most privileged in respondents’ academic environment are White, middle-aged, heterosexual men without care obligations and with good local, political, and informal connections. These findings show the potential of self-report measures to produce indications of relationality (e.g., where there is disadvantage, there is also favouritism and privilege; Atewologun and Sealy, 2014). In addition, the attributes that participants associated with being privileged and favoured in the academic workplace have little to do with academic skills and qualifications. Attributes that might be related more explicitly to one’s standing as an academic were named 21 times. However, 14 of those attributes are known to be gendered rather than based purely on merit, such as number of publications and the ability to attract funding. The same holds for the category “being seen by those in power,” which is more easily achieved when one is perceived as a “star academic” and “able to perform better than others,” which both may be consequences of belonging to “the inner circle” in the first place (Täuber and Mahmoudi, 2022). Together, this shows that self-report measures can also be used to create initial insights into social constructivism (Atewologun, 2018), which may then be followed-up and analysed in more detail by methodological approaches better suited to explore strong intersectionality.

### Limitations and Future Research

The research presented here largely aligns with prior research. But the relatively small sample size and the correlational nature of the data warrant some caution. In addition, although showing variance in intersectionality, the sample was still relatively homogenous and predominantly consisted of white women. Ideally, follow-up research would engage with larger and more heterogeneous samples. For policy-making, the suggested stepwise approach should be tested. Experimental studies might add insights about causality, allowing to better understand the associations between harassment, psychological safety, voice and career choice. In addition, future research might want to zoom in on the question what makes women in academia perceive policy as (in)effective. Here, policy ineffectiveness was operationalized as experiences of harassment, discrimination, retaliation after reporting, and institutional resistance to gender equality. Such experiences imply the ineffectiveness of anti-harassment policy and suggest inadequate complaint management. Another possibility would be to ask more directly about the perceived effectiveness of universities’ commitments and policies, interventions and measures.

Moreover, the self-designed construct of career choice was intended to measure expressions of discontent, not by speaking up, but by leaving academic working environments. One of the reviewers of this manuscript suggested, for instance, that contemplating changing careers could suggest agency, point towards practicing self-preservation or even reflect a form of self-empowerment by resisting toxic academic cultures. On the other hand, women academics who do chose to stay in their academic environment might have felt that being vocal about the culture would make them more vulnerable and open to retaliation, which could explain the lacking association between psychological safety and voice. These considerations suggest that follow-up research into voice, career choices, agency, and self-protection might be valuable.

### Practical Implications

The ineffectiveness of anti-harassment and non-discrimination policy, the inadequacy of complaint procedures, and the lack of successful interventions to increase gender equality in academia are not just unfortunate. Policy ineffectiveness can have adverse associations with relevant constructs, especially for women academics who differ from the majority on various dimensions. Policy is in part ineffective because it fails to account for intersectional experiences of inequality (Clavero and Galligan, 2021; Cortina and Areguin, 2021) that could inform the force field of power, privilege and systemic injustice in which policies are designed and implemented. Employing intersectional approaches to design and implement policy will benefit a wide range of academics. This is because, although the present research focused on women academics in particular, many of the experiences they shared involve gender harassment (Fitzgerald et al., 1995; Berdahl, 2007; Cortina and Areguin, 2021). Gender harassment affects everyone who deviates from gender stereotypes, including men of colour, identifying as LGBTQ+, with care responsibilities, or without a strong local and powerful network.

Besides the toll that policy ineffectiveness takes on individual academics, universities and society suffer from the resulting lack of perspectives and innovation. This is excellently documented by the diversity-innovation paradox in science described Hofstra et al. (2020). Based on data from the near-complete population of roughly 1.2 million US doctoral recipients from 1977 to 2015, the authors show that members of underrepresented groups produce higher rates of scientific novelty, yet find their novel contributions devalued and discounted. As a result, in comparison with majority groups, the innovations and novel contributions of scholars from underrepresented groups are less likely to translate into successful scientific careers. Implementing effective policies to tackle systemic inequality in academia thus benefits individual scholars from groups that are underrepresented and marginalized on multiple dimensions, as well as the higher education sector and society as a whole.

Finally, besides intersectional disadvantage, intersectional privilege needs to be more present in research and policy making. Women academics’ descriptions of the attributes someone needs to have in order to get ahead at their institutions show that intersectional privilege is seen very clearly. A stronger focus on those privileged in academia might be a useful complement to the more common focus on those experiencing disadvantage. The present research shows that the additive approach to intersectionality might offer a useful method to signal areas for further in-depth investigation. Ultimately, the mechanisms that maintain and reproduce inequality in academia can only be understood if privilege and power are on the research agenda—as a truly intersectional analysis would also suggest. The design of interventions and implementation of policies is often mandated to those benefitting from intersectional privilege, at the risk that unawareness of intersectional disadvantage and unwillingness to share power undermine the effectiveness of such measures.

## Conclusion

The present research shows that higher education institutions’ policy ineffectiveness contributes to the perpetuation and reproduction of inequality in academia, by driving especially groups out of the academy who are minoritized on multiple dimensions, while facilitating groups who are privileged on multiple dimensions. In the European context, it seems plausible that both lack of data and a weak, additive approach to intersectionality contribute to policy ineffectiveness. Equal and inclusive workplaces in higher education will not be achieved by relying on this co-optation approach. In this regard, much needed allyship for actual change must be achieved from those endowed with privilege and power by the unequal system that the higher education sector continues to be. Rather than having more workshops on unconscious bias and gender stereotypes, we need to have uncomfortable conversations about the power and privilege that is made possible for some by disadvantaging many others.

## Data Availability Statement

The raw data supporting the conclusions of this article will be made available by the authors, without undue reservation.

## Ethics Statement

Ethical review and approval was not required for the study on human participants in accordance with the local legislation and institutional requirements. The patients/participants provided their written informed consent to participate in this study.

## Author Contributions

ST conceptualized and designed the study, collected and analysed the data, and wrote the manuscript.

## Conflict of Interest

The author declares that the research was conducted in the absence of any commercial or financial relationships that could be construed as a potential conflict of interest.

## Publisher’s Note

All claims expressed in this article are solely those of the authors and do not necessarily represent those of their affiliated organizations, or those of the publisher, the editors and the reviewers. Any product that may be evaluated in this article, or claim that may be made by its manufacturer, is not guaranteed or endorsed by the publisher.

## Footnotes

^1^Sliwa and Johansson (2014) explain that their use of the label ‘foreign woman’ reflected “the most common descriptor mobilized by our participants in referring to themselves.” They further explain that they do not “apply the label ‘foreign woman’ in an essentialist sense.” Instead, they consider the label as an “umbrella term to describe a relational, heterogeneous category of workers, for whom depending on the situation and the individual, career trajectories will be influenced by different factors, not always affecting exclusively foreign women. Sometimes this might be gender alone, on other occasions the fact of belonging to a particular nationality or religious group, on yet other the intersection of, for example, gender, ethnicity and accent. What our research participants have in common is the fact of belonging to an organizational minority which is growing in numbers as the drive towards internationalization and globalization continues in United Kingdom higher education.”

^2^Note that the analyses presented here differ from the preregistration in a number of ways. First, the *a priori* power analysis was based on the expectation that the indicators of policy ineffectiveness would load onto one factor. The empirical data revealed that each loaded on a different factor, meaning that the power analysis should have been based on six instead of only three predictors. Further, the preregistration expected the components of the dependent variable Voice (voice and career choices) to load on one factor. The empirical data revealed that they loaded on different factors, resulting in two rather than one dependent variable. Together, the deviations from the preregistration warrant caution particularly with respect to the study’s power.
