# Supplementary material for: Women Academics’ Intersectional Experiences of Policy Ineffectiveness in the European Context
Source: Front Psychol. 2022 May 6;13:810569. doi: 10.3389/fpsyg.2022.810569 (PMC9122029; doi:10.3389/fpsyg.2022.810569)
Supplement: Supplementary file 1 [file Data_Sheet_1.pdf]

Appendix A - Questionnaire Items

Intersectionality (Athena Survey of Science, Engineering and Technology, 2016)

Please take a moment to consider the majority of colleagues at your institution. Do you differ from the majority? Please tick the boxes that apply to you. If you feel that you do not differ from the majority, please tick the last box.

- Race or ethnicity
- Country of origin
- Legal sex
- Age
- Sexual orientation:
- Disability status
- Religious affiliation:
- Social class
- Other dimension, namely (1)
- Other dimension, namely (2)
- Other dimension, namely (3)
- None, I do not differ from the majority of people at my work.

Intersectional disadvantage and privilege

Please rate the extent to which these dimensions have affected your career and career choices to date:

- My race or ethnicity:
- My country of origin:
- My legal sex:
- My age (relative to my peers):
- My sexual orientation:
- My gender identity:
- My disability status:
- My religious affiliation (or lack thereof):
- My caring responsibilities (or lack thereof):
- My marital or civil partnership status:
- Other dimension, namely (piped text inserted from first question):

Policy Ineffectiveness (Athena Survey of Science, Engineering, and Technology, 2016; Naezer et al. 2019; Svensson & Genugten, 2013; Flood et al., 2021)

Discrimination

In your main academic working environment, how common is resource allocation that favors men or other academics that are more similar to the majority group?

Appendix A - Questionnaire Items

- Allocation of desirable and sought-after tasks or roles
- Distribution of office space
- The receipt of mentoring and/or other guidance in making career decisions
- Representation in senior positions
- Allocation of administrative tasks
- Attention from senior management
- Access to informal circles of influence
- Receiving positive feedback from management
- Recruitment and selection for academic post
- Promotion decisions
- Allocation of formal training and career development opportunities
- Allocation of teaching
- Distribution of lab space or equipment
- Invitations to lectures, conferences, etc.
- Appointments to editorships of journals
- Recognition of intellectual contributions during meetings, conferences, workshops etc.

Privilege in resource allocation

In your academic environment, what kind of attributes would a person need to have in order to be most favored/ privileged in resource allocation?

- Attribute 1: …. Attribute 8:

Harassment

How common is it for you to experience or witness one of the following forms of workplace harassment during your academic career?

- Scientific sabotage
- Sexual harassment
- Physical and verbal threats, intimidation
- Being criticized unfairly
- Being excluded and marginalized
- Being characterized as having "special needs"
- Other form, namely:

Retaliation after reporting

Have you ever complained about harassment or discrimination? You can tick multiple boxes.

- Yes, I have complained about harassment or discrimination.
- I know other people who have complained about harassment or discrimination.

Appendix A - Questionnaire Items

- I have not complained about harassment or discrimination, neither I know people who have.

If you have ever complained about (gender) inequality at your organization, please indicate the extent to which you agree or disagree with the following statements. In case you have not complained yourself, please answer these items bearing in mind what you expect would be the most likely response in your academic environment.

- Being blamed for the situation.
- Being criticized for complaining about gender inequality.
- Being gossiped about in an unkind way.
- Being slighted or ignored by others at work.
- Being shunned or excluded by others at work.
- Being considered a “troublemaker”.
- Being threatened.
- Being given less favorable job duties.
- Being unfairly denied a deserved promotion.
- Being denied an opportunity for a deserved training.
- Being transferred to a less desirable job.
- Being given unfair poor job performance appraisals.
- If you have experienced any other form of retaliation, please share it here:

Institutional resistance to gender equality

In your academic working environment, how common are the following responses when issues of harassment or discrimination are brought up, either by yourself or others?

- Denial of the problem or the credibility of the case for change.
  Example: There is no problem here.
- Refusal to recognize responsibility.
  Example: It is not my job to do something here.
- Refusal to implement a change initiative.
  Example: It is not a priority right now.
- Efforts to placate or pacify those advocating for change in order to limit its impact
  Example: Yes, yes, we need to do something one day.
- Simulating change while covertly undermining it.
  Example: Of course, we’d appoint more women, if they were only more experienced.
- Using the language of progressive frameworks and goals for reactionary ends.
  Example: What about men’s rights? Men are victims too you know?
- Reversing or dismantling a change initiative.
  Example: We tried that once and women did not want to take up the promotion/training/ opportunity
- Aggressive, attacking response

Example: These feminists deserve all the abuse they get.

Psychological safety (Edmondson, 1999)

Please indicate how typical the following statements are for the academic environment in which you mostly work in:

- If I make a mistake, it is often held against me. (R)
- I am able to bring up problems and tough issues.
- People on this team sometimes reject others for being different. (R)
- It is safe to take a risk on this team.
- It is difficult to ask other colleagues for help. (R)
- No one of my colleagues would deliberately act in a way that undermines my efforts.
- Working with my colleagues, my unique skills and talents are valued and utilized.

Voice Attitudes (Liang et al., 2012)

Please indicate the extent to which you agree or disagree with the following statements:

- I proactively develop and make suggestions for issues that may influence gender equality in my working environment.
- I proactively suggest new projects which are beneficial gender equality in my working environment.
- I raise suggestions about gender equality to improve the unit's working procedure.
- I proactively voice out constructive suggestions concerning gender equality that help the unit reach its goals.
- I make constructive suggestions to improve the unit's operation.
- I advise other colleagues against undesirable behaviors that would hamper job performance.
- I speak up honestly with problems about gender inequality that might cause serious loss to the work unit, even when/though dissenting opinions exist.
- I dare to voice out opinions on gender inequality, even if that would embarrass others.
- I dare to point out problems about gender inequality when they appear in the unit, even if that would hamper relationships with other colleagues.
- I proactively report gender inequality problems in the workplace to the management.

Career choices (self-developed)

- I consider changing careers. (R)
- I consider changing departments/ universities. (R)
- I would recommend other women to work in academia.
- If I had a daughter, I would feel that academia is a good career choice/ is a safe working environment.

Appendix A - Questionnaire Items

If you would like to share any experiences or thoughts about the topic of (gender) equality, perceived resistance, harassment or discrimination, or complaint management, please feel free to do so here:

Questionnaire items as used in Täuber S (2022) Women Academics' Intersectional Experiences of Policy Ineffectiveness in the European Context. Front. Psychol. 13:810569. doi: 10.3389/fpsyg.2022.810569
